# Esophageal Cancer, the Topmost Cancer at MTRH in the Rift Valley, Kenya, and Its Potential Risk Factors

**DOI:** 10.1155/2013/503249

**Published:** 2013-12-29

**Authors:** Kirtika Patel, Johnston Wakhisi, Simeon Mining, Ann Mwangi, Radheka Patel

**Affiliations:** ^1^Department of Immunology, Moi University, Kenya; ^2^Department of Biochemistry, Moi University, Kenya; ^3^Department of Behavioral Science, Moi University, Kenya; ^4^Imperial College, London, UK

## Abstract

Esophageal cancer at Moi Teaching and Referral Hospital (MTRH) is the leading cancer in men with a poor prognosis. A case control study (*n* = 159) aimed at the histology type, gender, and risk indicators was carried out at MTRH. Mantel Haenszel chi-square and logistic regression were employed for analysis. Squamous-cell carcinoma was the common histological type occurring in the middle third portion of the oesophagus. The occurrence of the cancer in males was 1.4 times that of females. The mean age was 56.1 yrs. Low socioeconomic, smoking, snuff use, alcohol, tooth loss, cooking with charcoal and firewood, hot beverage, and use of *mursik* were independently associated with esophageal cancer (*P* < 0.05). Using logistic regression adjusted for various factors, alcohol consumption was associated with the increased risk of esophageal cancer. AHR was 0.45 and 95% CI: 0.205–0.985, *P* = 0.046. A societal component of low socioeconomic conditions, a lifestyle component with specific practices such as the consumption of *mursik, chang'aa, busaa*, snuff, smoking, hot tea, poor oral hygiene, and an environmental component with potential exposure to high levels of nitrosamines, passive smoking, and cooking with coal, could be involved. The increase in experts at MTRH capable of diagnosing could be responsible for the increase in reporting this neoplasm.

## 1. Introduction

Esophageal cancer is the eighth most common malignancy and sixth most common cause of cancer death worldwide [1]. It is one of the most deadly cancers with overall 5-year survival less than 10% [2]. An infamous “esophageal cancer belt” stretching from the north-eastern part of Iran to northern China has presented extremely high incidence of esophageal cancer, mainly squamous cell carcinoma (SCC) [3, 4]. South America, North America, Europe, India, and Southern Africa are other areas with high incidence rates of esophageal cancer [5–9].

Cancer of esophagus is a major and serious health problem in Sub-Saharan Africa [10]. It is a malignancy with a very poor prognosis. Esophageal cancer (EC) has striking geographic and sex variations in its incidence both regionally and worldwide. Western Kenya has a high rate of esophageal cancer [11–15]. The Moi Teaching and Referral Hospital (MTRH) is a tertiary level hospital, situated in the Rift Valley and has 800 bed capacity serving the population of Western Kenya. Cancer of the esophagus is the leading malignancy in males and only third to cancer of cervix and breast in females in terms of total cancer incidence reported at MTRH [13, 14]. The common symptoms of cancer of the esophagus are dysphagia and weight loss and by the time the patient presents for treatment the tumor is in the advanced stage and the prognosis is very poor.

It is proposed that the cause of this cancer is multifactorial. It is apparent that different etiological factors are at play in different high incidence areas of the world. Several possible predisposing etiologic factors could be responsible for esophageal cancer. These include the effect of deficiency of trace mineral deficiency [16], N-nitroso-compounds [17], contaminated food with mycotoxins [18], spicy food [19], tobacco smoking and alcohol [6], low socioeconomic status [20], familial nature of this disease [21], presence of infectious agents [22], and consumption of hot drinks [23]. Due to the wide geographical differences and substantial changes in the incidence of esophageal cancer over time, it has been suggested that these risk factors play a major role in the etiology of esophageal cancer [24]. This observation makes it desirable to study the features of esophageal cancer in different geographical settings. Besides, Kenya is widely regarded to have a richly diverse genetic pool with a reported 42 tribes in overlapping geographic regions proving to be a valuable source of research. Despite being described as a high-risk region for esophageal cancer in younger population, there have been few studies conducted in Kenya compared with other endemic areas.

Therefore, this study was undertaken to identify the risk factors in the population using a hospital-based case control study at MTRH. The results obtained from this study would help formulate the necessary steps to accomplish effective cancer care, screening, and prevention programs at MTRH.

## 2. Materials and Methods

159 patients who were diagnosed with primary ESCC at MTRH, Eldoret, Kenya, between June 2003 and July 2006 were recruited into the study. The set of controls (159) were obtained from MTRH, who were patients or relatives or visitors at the hospital with no relation to cancer. They were matched to the 159 cases (patients diagnosed with esophageal cancer) in relation to their area of residence, tribe, age (±2 yrs), sex, and time of admittance. A questionnaire was administered by research assistant to all cases and controls to demonstrate possible etiologic factors such as socio-economic levels, family history, occupational history, treatment sought before arriving at the hospital, alcohol consumption, tobacco consumption, cigarette smoking, nutritional deficiencies, consumption of hot drinks, and food and other drinks consumed, chillies, exposure to chemicals and polycyclic aromatic hydrocarbons during cooking and sleeping, various food consumed, rate of intake of food, tooth loss, and HIV status associated with the appearance of esophageal cancer. Ethical permission to carry out the study was obtained from the Institutions Research and Ethical Committee (IREC). All patients involved in this study signed a written consent to participate in this study.

## 3. Results and Discussion

A case-control study comprised of 159 newly diagnosed cases of cancer of the oesophagus and 159 controls were seen at MTRH from the period of June 1, 2003 and July 31, 2006. 159 cases of esophageal cancer recorded during the study period accounted for 7.15% of the total 2222 neoplasms at MTRH. Squamous-cell carcinoma (SCC) accounted for 92.45% (147 cases), while adenocarcinomas (ADC) accounted for  7.54% (12 cases) from the total of 159 cases. The preponderance of SCC over ADC and the late tumor state at the time of presentation are in agreement with virtually all other published reports from other endemic region [25, 26]. SCC developed in the middle third of the organ of 57.86% (92 cases), 31.44% (50 cases) occurred in the lower third, and 10.8% (17 cases) occurred in the upper third of the esophagus. The middle third of the esophagus was the most common location for tumors in this study. SCC developed in flat cells that lined the esophagus. Similar pattern of esophageal cancer has been reported [27, 28].

### 3.1. Demographic Characteristics

The number of males who had esophageal cancer in this study was 92 (57.9%) and females was 67 (42.1%). The male to female ratio was 1.4 : 1. Both sexes had the likelihood of getting cancer of esophagus in a similar proportion. These results indicate that the risk factors for male and female would be similar and linked to environment or life-style component. Hormonal factors may not play a role in the causation of esophageal cancer. The mean age for males was 56.09 years (SD 15.1) and the median age was 59 years, with the range of 12–98 years. The corresponding mean age for females was 55.03 years (SD 16.6) while the median age was 54.5 years with a range of 15–98 years. This finding was significant and similar to an earlier study conducted [15] in the Rift Valley. This demonstrated that there was consistency in esophageal cancer based on sex from two different areas in the Rift Valley. Other studies have reported that sex ratio was reported to be 26.5 : 1 and 6.0 : 1 in Johannesburg [25]. In both of the studies women remained the least affected by esophageal cancer. In addition, studies in Uganda came up with a ratio of 2 : 1 in favour of women [26]. It has been argued that in the developed world the ratio was 5 : 1 as men drink alcohol and smoke more than women [29]. The mean age was 56.09 years (SD 15.1) while the median age was 59 years. The youngest patient was 12 yrs old and the eldest was 98 yrs old. This was consistent with the published report in Tenwek Hospital, Bomet District, in Western Kenya [12, 15]. This study and the Bomet study are the only two reports showing esophageal cancer in very young population below 20 yrs. This finding was alarming as it seems that the population, in the area of this study, has younger patients with esophagus cancer compared to other high-risk areas where esophageal cancer in under 20 yrs was below 1% [29, 30].

It was observed during this investigation that vomiting, hematemesis, chest pain dysphagia, and weight loss were the most common presenting symptoms for cancer of the oesophagus. The observations were however not peculiar as they appear to be common symptoms among patients with esophageal cancer [27, 31].

The distribution of patients with esophageal cancer was seen from districts near the hospital, for example Keiyo, Uasin Gishu, and Nandi, and referrals from health clinics in close vicinity to MTRH. The distribution by tribe for both case and controls were *n* = 82 (51.9%) *Kalenjin*, *n* = 45 (28.5%) *Luhya*, *n* = 13 (8.2%) *Kikuyu*, *n* = 9 (5.7%) *Luo*, *n* = 4 (2.5%) *Turkana*, and other tribes* Kambas and Kisii *and Sudanese *n* = 5 (3.2%) which represented the normal distribution of the population living in the study area. However, it did not imply that the *Kalenjins* were the only group predisposed to cancer of the esophagus. In the Rift Valley province the highest number of patients 21.9% (*n* = 25) came both from Uasin Gishu North and Keiyo district. Since MTRH, where the studies reported here were conducted, is located in the Uasin Gishu district the foregoing observation was expected. However, it was very interesting to record that in spite of such a high number of patients from Keiyo district there was low number of biopsies obtained from them and it could be due to the belief among them that if one went to the hospital for any surgery one would not come out alive. Oncologist, from Nairobi and the coastal area of Kenya both with different tribes have experienced a rise in esophagus cancer [32]. This could also be explained by, presence of experts that has raised the awareness of this “already existing” problem.

### 3.2. Socioeconomic Status

#### 3.2.1. Education and Monthly Income

In terms of level of education primary, secondary, and tertiary were studied. Controls (*n* = 92) had more educated candidates at secondary and tertiary levels than the cases (*n* = 60) with a statistically significant association (*P* < 0.05). The economic status was calculated based on monthly income either less than or more than $1000/- per month. There was a statistically significant association (*P* < 0.001). The observed odds ratio (OR) was 0.593 and the 95% confidence interval (CI) for the odds ratio was (0.458–0.768). The results implied that cases were economically not so well off than the controls by 40.7% (1–0.593). The low socio-economic status has been known to be an important factor in the causation of esophageal cancer in the majority of the studies [33, 34]. Information was not obtained from 12 (7.2%) of the cases and 6 (3.8%) of the controls as they had difficulty in answering this question.

#### 3.2.2. Housing and Roofing, Number of Occupants and Items Owned, and Source of Water

The housing in this study population varied from multifloor brick house to mud and plant material houses. The cases and the controls were statistically homogeneous (*P* = 0.101) in respect of the type of housing. Grass or straw was more common than iron sheeting as roofing material, with cases, indicating their low socio-economic conditions as compared to controls. There was a significant association between cases and controls with a *P* value of < 0.001. The OR was 0.338 and 95% CI was (0.227–0.502). The results showed that patients with esophageal cancer had 33% more likely to have grass thatched houses compared to patients that do not have esophageal cancer. Grass thatched houses also encourage inhabitation of fungi and bacteria which could be a risk factor for infection. Data accumulated on the size of the total living area occupied by the participants. The cases had smaller pieces of land compared to controls, indicating their low socio-economic condition. There was a statistical association in land ownership with respect to the two groups where *P* value was *P* < 0.001 and hence the difference between cases and control was very significant. The majority of the cases (93.1%) owned less than 0.25 acres, when compared to 56% of controls who owned less than 0.25 acre or less. The OR was 10.6 with 95% CI (5.1–22.4). This result showed that cases were 10.6 times less likely to own higher acreage of land than controls. Data indicated that the number of house occupants ranged between 2 and 15 with no significant difference. The OR was 1.009 with 95% CI of (0.623–1.052). The data reveals that cases and controls are different in terms of ownership of bicycle and cars. 66.3% of the controls own bicycles, and 75% own cars. In the case group, 59.5% do not own any of the items listed compared to controls, where 37.7% did not own any items. This suggests that people with esophageal cancer own less items and are of lower socio-economic level compared to controls. At 5% level of significance, the data statistically supports difference in proportions of household items in the case and control populations with *P* = 0.003. The OR was 1.273 with 95% CI (0.983–1.650). The data suggest that cases owned 1.3 times less items compared to controls which made them socioeconomically less affluent compared to controls. The range of the source of water used by the study participants varied from natural source to piped water in the house. Cases and controls used the natural source of water. There was no statistically significant difference between the two groups *P* > 0.05. Both groups used more than one source of water therefore giving multiple responses. The population in this study relied on natural sources of water. However, the ground water they used was not analysed for the presence of carcinogens in this study. It was thus not immediately possible to establish if there were any water pollutants and whether they contributed in any way to the causation of esophageal cancer. Water analysis in Marsabit in Northern Kenya has shown that toxic chemicals like nitrates, nitrites, and arsenic were found in water and were linked to incidence of cancer of the esophagus reported in that region [35].

### 3.3. Choice of Hospital, Occupation, Exposure to Other Chemicals, and Cooking Fuel Used

Referral hospital was the first choice for patients with esophageal cancer compared to the controls. This is not astonishing as patients with esophageal cancer have a difficulty in swallowing food and that requires immediate attention by experts. Esophageal cancer is a severe disease with mortality rate of less than 5 years in Western countries [36]. The surprising fact was that in spite of the severity of disease there were 29.6% (47 cases) that did not come to the hospital early enough and sought help elsewhere specially with traditional healers as seen. Statistically, there was an association between the two groups with *P* < 0.001 and OR of 0.66 with 95% CI (0.533–0.836). This implied that the control population were 66% less likely to seek treatment at referral hospital than a patient with esophageal cancer as diseases suffered by controls were not as severe as cases. There is a community fear for hospitals and belief that going to hospital for surgery would lead to death.

The common occupation was farming and in cases of women it was housewives in both groups of cases and controls, which is not surprising as the study population came from an agricultural area.

Cases and control populations are statistically different, (*P* < 0.05) with respect to their use of various chemicals. In the population of the cases, 67.3%, 54.8%, and 53.4% use insecticides, detergents, and cleaning liquids, respectively. For the control population, 80.5%, 60%, and 57.9% use garden sprays, printing ink, and paint/asbestos/solvents, respectively. This may have implication that insecticides used may be a risk factor for getting esophageal cancer. However, a study carried out on the use of insecticides and esophageal cancer did not find any correlation [37]. This aspect of the study requires further research in the high incidence area.

A range of fuel products ranging from firewood, charcoal, kerosene, cooking gas, and electric plates were used. In this study, firewood and charcoal were grouped in one and other fuel products in the other group. Regarding cooking fuel, 70 (44.4%) of the cases and 41 (25.8%) of the controls reported to cook with charcoal and firewood. There was significant difference with cooking with charcoal and firewood between cases and controls and esophageal cancer in this study (OR 2.32). Indoor air pollution from charcoal burning is a known human carcinogen [38], while that from biomass (primarily wood burning) is a probable human carcinogen [38, 39] It contains many hazardous pollutants including known human carcinogens such as benzo(a)pyrene, formaldehyde, and benzene [39]. Exposure to such indoor air pollution is a major public health concern in Kenya because the majority of its population still relies on natural fuels for cooking. A study in China and India showed that indoor air pollution from charcoal burning is a risk factor for esophageal and hypopharyngeal cancer [40, 41]. This may suggest that long-term exposure to indoor smoke created with cooking with charcoal and firewood may be a risk factor for esophageal cancer incidence among the lower socio-economic population which rely on these natural sources for cooking fuel and sleeping in the same room where they cook.

### 3.4. Smoking and Other Substance Used

In this study it was revealed that tobacco smoking had a role in the aetiogenesis of esophageal cancer. The cases had 2.51 times more chances of getting esophageal cancer than the controls (OR 2.51) if they smoked. The tar amount of cigarette has been seen to have a positive correlation with esophageal cancer [42]. The increased risk of black tobacco compared to blond tobacco in causation of cancers of upper aerodigestive tract in southern Europe and Latin America is high [43]. The results are consistent with other studies carried out in high-risk areas of South America [30, 44]. Most of the cases in the smokers group (35/44) used hand rolled cigarettes that used black tobacco. Hand rolled cigarettes contain high yields of benzopyrene and benzene which are proven carcinogens [30].

Other substance uses like *Miraa* and *Bhangi* and Khat were not significant risk factors (*P* = 0.188) compared to controls in this study. In order to explore the joint effects of alcohol and tobacco and other substances, another study with interaction tests should be carried out. There was evidence that the effect of both exposures was nearly multiplicative. With multiplicative effect, the risk of being simultaneously a heavy drinker (≥75 g/d) and heavy smoker (>50 sticks/d) could be as much as 80% [30]. Alcohol acts as an initiator while smoking and other substance use acts during later stages as a promoter in the development of esophageal cancer [30].

Snuff has reduced levels of carcinogenic tobacco-specific nitrosamines as compared to cigarette. The use of snuff therefore can help smokers to stop or reduce their smoking habits. In this study, there was a relationship seen with the use of snuff and esophageal cancer (OR 4.74) as also seen by other researchers [45]. Other studies did not show any relationship between snuff and esophageal cancer [46, 47]. There was a limitation as the number and power had insufficient covariate information to rule out important positive or negative confounding by smoking intensity.

### 3.5. Alcohol

It was revealed in this study that the consumption of alcohol appeared to have a role to play in the aetiology of esophageal cancer among the local residents (OR 2.64). It was observed in this study that alcohol drinking was a much stronger risk factor for cases with squamous-cell carcinoma than for the whole control series. Alcohol was mainly consumed in the form of local brew. The local maize brews which were commonly consumed were *chang'aa *(vodka) and *busaa* or* maiyek* (millet beer) and *kumik* (honey beer). This study shows that there was a significant difference between the cases and controls (*P* < 0.001) in alcohol consumption and the odds ratio (OR 2.64) shows that cases were 2.64 times more likely to get esophageal cancer than control due to alcohol consumption.

The amount and the duration (in years) of alcohol consumed by cases and the controls were also significant in this study. Intensity of alcohol drinking has been found to be a more relevant predictor of risk than the duration of the habit. The age at which subjects started drinking was not associated with risk and the cessation of drinking was not associated with any beneficial effect even ≥5 y after cessation. A beneficial effect has been found in some studies particularly 10 years after giving up drinking [47–49] although some studies have shown a rapid decline in risk after cessation of drinking [50–52]. On the contrary, other studies have shown either a nonbeneficial effect [53] or a higher risk among former drinker [51].

This study also reveals that the cases and controls have different preference towards various types of alcohol. The cases preferred local brews which had a high alcohol content compared to other drinks probably due to their socio-economic condition as local brews are much cheaper. An ingredient common to all beverages is ethanol although it is possible that other components or contaminants such as N-nitrosamines and urethane with carcinogenic properties may increase cancer risk. It has been shown in practically all the studies that the risks are the greatest for drinkers of hard liquors which is consistent with evidence that the concentration of ethanol plays an important role in alcohol-related tumours of the upper aerodigestive tract [54]. Thus, it has been suggested that in addition to a systemic effect, ethanol can be converted to acetaldehyde in saliva and exert a promoting effect by either solubilising tobacco-specific carcinogens or enhancing their penetration into the esophageal mucosa, by nutritional deficiencies associated with heavy drinking or by other mechanisms (direct toxic or oxidative effect on the epithelial mucosa) [55]. Beers made from maize and millet may also have fungal contaminants. In Transkei, fungal contaminants like yeast and fumonisins were shown to play a part in the aetiology of esophageal cancer [56].

There were two cases that had alcohol for more than 41 yrs. Both cases came from Keiyo district. It has been documented by Chebet and Dietz (2000) [57] that the people from Keiyo are very fond of a local brew *maiyek (millet beer)* and *kumik (honey beer)*.

It has been also suggested that part of the effect observed for alcohol and/or tobacco smoking could be due to other factors or different lifestyles, such as a low fruit and vegetable intake [58]. However, the data was not adjusted for the intake of fruit and vegetables showing that alcohol and tobacco were strong risk factors although the risk estimates should have been much higher if adjusted for fruits and vegetables intake.

### 3.6. Meals and Beverages and Tooth Loss

Meals consumed was mainly maize meals, wheat chapattis, seasonal fruits and vegetables, and red and white meat. The inhabitants of the study area typically consume maize at every meal. Maize is a poor source of B-complex vitamins, particularly riboflavin and niacin [59]. Deficiency of such vitamins results in mucosal inflammation [60] which may predispose the epithelial tissue to malignancy. Maize meal therefore may predispose to endemic squamous cancer of the oesophagus in the study area and maybe in wider regions in the African continent. An important factor is the breakdown of esterified linoleic acid to the free form in stored maize meal. This is known to lead to excess production of prostaglandin E2 in the stomach. The excess prostaglandin E2 causes a low-acid duodenogastro-esophageal reflux, which predisposes to carcinogenesis [61]. Another possible explanation proposed that the reliance on maize increased exposure to the mycotoxin fumonisin produced by the commonly occurring maize mold *Fusarium moniliforme* [62]. Fumonisins in association with nitrosamines have been shown to be correlated with the prevalence of esophageal cancer in the Eastern Cape, a high incidence area of esophageal cancer in Southern African, [62] and in China [18] and may be rampant in other maize consuming regions of the world. Weekly intake of meals was less in patients with esophageal cancer (cases) than with people without esophageal cancer (controls) with a statistical significance at *P* < 0.001 and OR 0.37 with 95% CI of OR was (0.22–0.62). The results imply that the cases were 37% more likely to have fewer meals per week than controls. The cases were from lower socio-economic background and would have found it difficult to have regular balanced diet which is very important to health. Failure to take a regular balanced diet can be a predisposing factor to cancer. Big and hard bullous of food with rough edges taken in fast causes mechanical damage to esophageal lining thus causing inflammation and can be a predisposing factor to esophageal cancer. There was a significant difference between the way food was taken by both cases and controls *P* = 0.023, OR was 0.81 with 95% CI for OR (0.5–1.3). The results imply that cases eat much slower than controls. However, a bias cannot be ruled out as patients suffering from esophageal cancer have difficulty in swallowing food. High temperature of food taken can cause thermal injury. There was a significant difference between the cases and controls in the preferred temperature of food intake before the onset of the disease as the *P* < 0.001 OR 12.3 and the 95% CI for OR was (6.5–23.6). The cases were 12.3 times more likely to have esophageal cancer if they had very hot food compared to the controls. There was no significant difference between the consumption of various food items between the cases and controls *P* = 0.396 OR 0.7 and 95% CI of OR was (0.28–1.73). The statistical calculation shows a low OR and high *P* value and therefore not significant difference of what the cases and controls consumed. Contaminated maize has been known to be a risk factor predisposing to esophageal cancer but it was beyond the scope of this study to assess the contamination. Red meat and specially *Nyama choma* was also known to be a risk factor predisposing to esophageal cancer. It was beyond the scope of this study to assess the quantity and frequency of the above variable. Food containing chillies have been known to be a predisposing factor to esophageal cancer. Chillies or spicy food was not taken by both the cases and control in this study population. In the current study it was observed that residents in the study area frequently consumed green leafy vegetables specially *Kale *also locally known as *Sukuma wiki*. In Transkei a green leafy plant weed, *Solarium nigrum* [56], and in the Kashmir valley in India, *Brassica oleracea* (“*Hakh*”) cruciferous leafy vegetables [8] consumption was associated as risk factors of esophageal cancer due to high percentage of nitrosamines content in leafy vegetables. It would seem that further studies are required especially in the biochemical analysis of *Kale* to verify if it contained ingredients such as nitrites and nitrates which could precipitate cancer of the oesophagus.

In this study population ingestion of red meat cooked at high temperature directly on charcoal (*Nyama Choma*) and smoked and salted food was very common. These foods contain nitrosamines that could be attributed to a predisposing factor that the population was exposed to. From evidence in other studies, the nitrosamines are known to be carcinogens that cause genetic mutation by DNA alkylation [63, 64]. Heterocyclic amines, benzopyrene, and polycyclic hydrocarbons formed in meat cooked at high temperatures or open flame are linked to esophageal cancer [65–67]. Dietary habits that may increase the risk of esophageal cancer are inadequate mastication of food, eating hard and scratchy food, highly salted food, and smoked meat or fish [68].

Patients with esophageal cancer (cases) had a preference to hot beverages like hot tea or hot *uji*. In this study it was seen that hot drinks were preferred by the cases as compared to the controls before the onset of illness (OR 12.78). Hot drinks were associated with a higher risk of esophageal cancer in a recent study in Iran [69]. Epidemiologic studies describing the association were conducted in areas with a high incidence of esophageal cancer where consumption of hot drinks (maté) is popular. Drinking hot maté is classified as possibly carcinogenic according to IARC [70]. The pathogenic pathway can be dual; consumption of maté can act as a carcinogen or promoter itself, or the high temperature of the drink alone is responsible for carcinogenesis. It is thought that the high temperature causes esophageal cancer through repeated thermal injury [71]. Since the aetiology of cancers is multifactorial, drinking hot drinks and food, smoking, consuming alcohol, and insufficient consumption of fruits and vegetables must all be taken into account [72].

*Mursik* a popular drink with people from this area was consumed more by patients with esophageal cancer than controls. An association with drinking of “*mursik*” as a risk factor was seen in this study (OR 3.72).* Mursik* is fermented milk laced with charcoal powder and is very common drink for the local population. “*Mursik*” is a local beverage which is likely to be contaminated with polycyclic aromatic hydrocarbons (PAHs) as it contains some particles of charcoal from a special herb plant. The charcoal is ground and added to the container to flavour the milk. Polycyclic aromatic hydrocarbons (PAHs) are known to be carcinogenic agents. Ingestion of food contaminated with household smoke or soot particles has recently been suggested as a possible route of esophageal exposure to PAHs and was common in the high-risk areas of Northern China [73]. An additional factor, which may interact with this exposure, is that in people with genetic variation in the enzymes responsible for the metabolism of PAHs there is increased risk of developing squamous dysplasia of the esophagus which may be a predisposing factor to esophageal cancer [74]. *Mursik *has high levels of acetaldehyde and may thus contribute to esophageal carcinogenesis [75].

In this study tooth loss between cases and controls was significant (OR 5.28). Recent study showed a significant positive association between tooth loss and the risk of esophageal cancer. The findings indicate that preventive efforts aimed at the preservation of teeth may decrease the risk of these cancers [76]. The link maybe because tooth loss is a common consequence of chronic bacterial infection and may, therefore, serve as a surrogate for chronic infection and inflammation, which in turn may be important to the pathogenesis of cancer. Furthermore, persons who have lost teeth may not be able to eat a healthy diet, and diet is also a factor in cancer.

### 3.7. Family History of Cancer and HIV Awareness

Other cancers like esophageal, breast, cervix, stomach, blood, and kidney are prevalent in families of both cases and controls. Two families had a father and son both suffering from esophageal cancer. It shows that there is emergence of various cancers in this region. Due to lack of awareness and taboo associated with cancer in the study population the number of cancer could be an under representation.

*Awareness of HIV Status of Patients with and without Esophageal Cancer*. There was no significant difference reported between HIV status and esophageal cancer between cases and controls. At 5% level of significance *P* = 0.132. The results were based on the patient's answers to the questions in the questionnaire and HIV testing was not performed. A bias could have been introduced in the study as HIV had stigma attached to it especially in the age group questioned in this study.

## 4. Summary: Risk Factors Predisposing to Cancer of Oesophagus

Table 1 summarises the potential risk factor associated with esophageal cancer in this study. Smoking, snuff use, alcohol, loss of teeth, cooking with charcoal and firewood, taking hot beverage, and use of *mursik* were independently associated with esophageal cancer. At 5% level of significance all were significant (*P* < 0.05).

In a multivariate logistic regression, adjusting for gender, age, smoking, snuff use, and cooking and sleeping in the same room, alcohol consumption was associated with increased risk of developing oesophagus cancer and OR = 0.449 and 95% CI of the OR was (0.205–0.985). At 5% level of the difference significance was *P* = 0.046. After removing the above confounding factors the cases were 45% more likely to have esophageal cancer due to alcohol as compared to controls.

## 5. Conclusion

Squamous-cell carcinoma was the most common histology of esophageal cancer occurring in the middle third portion of the oesophagus. The male to female ratio was 1.4 : 1. The mean age was 56.1 (SD 15.1).

Esophageal cancer in the Rift Valley is the consequence of a multifactorial process. The three main component, emerge as important factors**:** *a low socio-economic* component with effect on education general living conditions like housing and food and nutrient intake; *a lifestyle* component with specific cultural practices such as consumption of *mursik, chang'aa, Busaa*, snuff, hot tea, and tooth loss; and *an environmental* component with potential exposure to high levels of dietary nitrosamines from diverse sources like smoking and use of coal for cooking. Using logistic regression adjusting for gender, age, smoking, snuff use, and cooking with charcoal and firewood, alcohol consumption was associated with increased risk esophageal cancer.

## Figures and Tables

**Table 1:** Summary: risk factors predisposing to cancer of oesophagus.

Variable	Cases (%)	Control (%)	OR	95%CI	*P* value
Smoking					
Yes	44 (27.7)	21 (13.2)	2.51	1.36–4.66	0.001
No	115 (72.3)	138 (86.8)			
Snuff use					
Yes	9 (5.7)	2 (1.3)	4.74	0.94–32.32	0.031
No	149 (94.3)	157 (98.7)			
Alcohol					
Yes	62 (39)	31 (19.5)	2.64	1.55–4.52	<0.001
No	97 (61)	128 (80.5)			
Tooth loss					
Yes	75 (47.2)	23 (14.5)	5.28	2.98–9.41	<0.001
No	84 (52.8)	136 (85.5)			
Cooking					
Yes	41 (25.8)	71 (44.4)	2.32	1.41–3.84	<0.001
No	118 (74.2)	88 (55.6)			
Take hot beverage					
Yes	139 (87.4)	56 (35.3)	12.78	6.98–23.6	<0.001
No	20 (12.6)	103 (64.7)			
Use *mursik *					
Yes	49 (30.7)	17 (10.5)	3.72	1.96–7.14	<0.001
No	110 (69.3)	142 (89.5)			

CI: confidence intervals; OR: odds ratio.
